# Channel-Hopping Sequence and Rendezvous MAC for Cognitive Radio Networks

**DOI:** 10.3390/s22165949

**Published:** 2022-08-09

**Authors:** Rajib Paul, Jiwoon Jang, Young-June Choi

**Affiliations:** 1 Department of Software and Computer Engineering, Ajou University, Suwon-si 16499, Korea; 2 Department of Information Technology, Ulsan College, Dong-gu, Ulsan 44022, Korea

**Keywords:** cognitive radio networks (CRNs), blind rendezvous, MAC protocol, probe request/response

## Abstract

In cognitive radio networks (CRNs), two secondary users (SUs) need to meet on a channel among multiple channels within a finite time to establish a link, which is called rendezvous. For blind rendezvous, researchers have devised ample well-grounded channel hopping (CH) sequences that guarantee smaller time-to-rendezvous. However, the best part of these works lacks the impact of network factors, particularly channel availability and collision during rendezvous. In this study, a new CH scheme is investigated by jointly considering the medium access control (MAC) protocol for single-hop multi-user CRNs. The analysis of our new variable hopping sequence (V-HS) guarantees rendezvous for the asymmetric channel model within a finite time. Although this mathematical concept guarantees rendezvous between two SUs, opportunities can be overthrown because of the unsuccessful exchange of control packets on that channel. A successful rendezvous also requires the exchange of messages reliably while two users visit the same channel. We propose a MAC protocol, namely ReMAC, that can work with V-HS and CH schemes. This design allows multiple rendezvous opportunities when a certain user visits the channel and modifies the conventional back-off strategy to maintain the channel list. Both simulation and analytical results exhibited improved performance over the previous approaches.

## 1. Introduction

Cognitive radio networks (CRNs) [1] have received considerable attention from the research community because of their ability to unfold the opportunistic use of the overly crowded spectrum. In the light of CRNs, we can employ a new communication standard that is more intelligent and flexible than conventional communication systems. Cognitive radio (CR) technology adopts a dynamic spectrum access (DSA) mechanism, which increases the benefit of the underutilized spectrum. Primary users (PUs) are the leading customers of the licensed spectrum, and secondary users (SUs) use DSA to detect unoccupied spectrum and exploit spectrum opportunities. SUs restore interference-free communication by leaving the spectrum if the PU reclaims them. Hence, PUs and SUs can coordinate in a distributed CRN by considering the dynamic channel availability.

In general, SUs sense an idle or free channel and access the channel for communication. When an SU senses a channel free from PUs, the channel is listed for rendezvous attempt. The number of available channels for each SU changes dynamically because the presence of a PU changes with frequency, time, and space. Thus, SUs can operate independently on different channels at any given time. However, communication is only possible when two SUs obtain a common channel between them and exchange control information. This fundamental issue is referred to as rendezvous [2,3]. In traditional wireless networks, a good practice is to preserve a common control channel (CCC) for negotiation between SUs. Many distributed MAC protocols [4] have adopted CCC to solve the rendezvous problem for the sake of simplicity. Rendezvous becomes simple if a CCC is present [5,6,7]; however, it has several disadvantages, such as control channel congestion, vulnerability to attack, and dynamic behavior of channels. Multiple CCCs have also been proposed by researchers; however, they increase the overhead of the networks and reduce the number of data channels.

In the light of such limitations, without depending on the CCC, a blind rendezvous [3] is preferred. Therefore, in recent years [8], many intensive studies have been reported on channel-hopping (CH)-based rendezvous as an ideal technique to solve the problem of blind rendezvous. The basic idea is to hop on a channel in each slot by following a sequence from a prearranged channel list and ensure that any two SUs meet on the same channel. The number of channels between any two users can be the same or different, that is, symmetric or asymmetric, respectively [9]. The performance of a CH sequence is often evaluated using time-to-rendezvous (TTR), that is, the number of slots needed by an SU go through before hopping on the same channel with another SU. The primary targets are to minimize the expected TTR (ETTR) and maximum TTR (MTTR).

In this paper, we propose a variable channel-hopping scheme, V-HS, that guarantees rendezvous in both symmetric and asymmetric models. The fundamental idea of the scheme is that during even time slots in a given time, SUs hop on different channels, whereas during odd time slots, SUs always stay on the same channel for rendezvous. For the symmetric model, we theoretically prove that V-HS guarantees rendezvous within 2P, which is less than 3P of the jump-and-stay (JS) algorithm [10], where *P* are the smallest prime number greater than the number of channels. For the asymmetric model, we also derive the upper bound of TTR, 4P(P−G+1), where *G* is the number of common channels between two users. The present study is the first to generate a hopping sequence that guarantees a rendezvous for asymmetric channel lists. In this study, we also obtained the expected TTR of V-HS for both models and confirmed via numerical results that V-HS outperforms JS.

For these hopping sequences, one underlying assumption is that two users achieve rendezvous as long as they visit the same channel simultaneously. However, this is not true in reality because it is not guaranteed that the messages exchanged between the two users are delivered reliably because of noise, interference, or collision, which are inherent problems in wireless communication. If any two SUs tend to communicate with each other, the first requirement is to find each other on the same channel at the same time slot and exchange control information without interrupting the PUs. Therefore, the existing hopping algorithms need to work under appropriate MAC protocols to guarantee successful rendezvous in CRNs. A multi-channel MAC protocol is appropriate to provide protection for the PUs by changing the channel list based on the activity of the PUs and enhancing spectrum usage.

In this paper, an active scanning-based rendezvous MAC (ReMAC) protocol is proposed for CRNs that can minimize the rendezvous time. The proposed ReMAC does not require a CCC to exchange control information. Similar to the 802.11 scanning process, the system is time slotted, and each slot is sufficiently large for two SUs to exchange control messages. However, there is a possibility of unreliable delivery of control messages; that is, rendezvous is not achieved, although the two SUs visit the same channel simultaneously. In particular, when there are many users to rendezvous, collisions between control messages or any other interference may occur. To deal with such cases, we adopt the collision resolution procedure of carrier-sense multiple access with collision avoidance (CSMA/CA) in the IEEE 802.11 MAC protocol to increase the back-off window size per failure.

Furthermore, ReMAC enables multiple rendezvous opportunities even in a slot when users visit a certain channel. If the slot size is small, they may transmit the control message only once. However, considering the channel-switching overhead between consecutive slots, several rendezvous opportunities are possible, that is, requests and responses are sent multiple times within a slot until they are successful. This is the first study to reveal multiple rendezvous opportunities in a slot when designing a rendezvous MAC protocol. Our ReMAC incorporates the proposed hopping sequence and maintains the integrity of the rendezvous. The contributions of this study are summarized as follows:

(1) We proposed a new channel-hopping sequence, V-HS, that guarantees rendezvous for the symmetric and asymmetric models. The channel list includes the number of available channels and channel rank for the user. Further, we analyzed a theoretical framework with a closed-form expression to guarantee rendezvous.
(2) We designed a new rendezvous protocol, ReMAC, based on probe request and response that is integrated with any channel-hopping sequence. This mechanism increases the probability of a successful handshake when two users come to the same channel. It resolves reliable transmission issues, such as collision; we tailor part of the IEEE 802.11 MAC protocol appropriately with the proposed rendezvous scheme.
(3) We compared our proposed V-HS and ReMAC under the constraints of collision and without collision with several state-of-the-art CH rendezvous schemes. Subsequently, we demonstrate that ReMAC can resolve the control packet collision and, therefore, outperforms the existing methods in terms of TTR.

The major focus of this study is to establish a link between two SUs, thereby guaranteeing rendezvous on a single channel at a time. Therefore, some of the follow-on tasks, such as channel contention [11], data packet transmission [12], optimal slot size for rendezvous [13], and multi-interface multi-hop rendezvous [14] are beyond the scope of this study.

The remainder of this paper is organized as follows. Section 2 presents related works. The system model and parameters are explained in Section 3. Subsequently, in Section 4, we propose a new hopping sequence, V-HS, and analyze the maximum and expected TTR of V-HS for both symmetric and asymmetric models. In Section 5, we propose a new rendezvous MAC protocol, ReMAC, which handles collision resolution and multiple rendezvous opportunities and derives the expected TTR in the MAC layer. Simulation results are presented in Section 6, followed by the conclusion in Section 7.

## 2. Related Works

Several extensive studies have been conducted on the rendezvous process. Most of these rendezvous protocols were originally inspired by traditional wireless networks. An efficient rendezvous process should provide guaranteed initialization, robustness, protection of PU transmission, and fairness for all SUs. However, the current rendezvous schemes do not support all these characteristics. The existing rendezvous taxonomy consists of two branches: aided and unaided systems. In an aided system, a central controller directs the users in the network. It is the controller that determines the available, possible links, and transmission schedule of a user. If a CCC is assigned, the system becomes highly vulnerable to attacks and is less scalable for a large number of users. Therefore, an aided system can create a network with a single point of failure.

In the unaided rendezvous system, users need to find the spectrum by their own account. The most easily applied scheme is to have an already known CCC to exchange control messages for negotiation between the users. The concept of CCC is employed by many distributed MAC protocols because rendezvous is achieved easily by having a CCC to help gather information about their neighbors and the network. There are two categories of CCC [6,7]: (i) global CCC and (ii) local CCC. In the former, a predefined control channel is assigned for all the users in the network. However, it is not guaranteed that there is a CCC that covers the entire network because users can have a different available channel set depending on the location or network environment. In addition, the overhead caused by collisions of control packets on the CCC may be severe because of the large number of users. For this reason, IEEE 802.22 avoids CCC and instead selects a channel from the available spectrum holes [15]. In contrast, the latter strategy allows multiple CCCs for each group or cluster. However, cluster establishment and communication overhead among clusters in the network are additional problems that need to be resolved. Moreover, both CCC schemes have a critical drawback, called a single point of failure. If the CCC is unavailable owing to a PU or jamming attack by malicious users in the network, the entire network initialization will fail.

According to [16], the fundamental concept of DSA does not acknowledge the requirement of a CCC, although some implementations suggest the opposite [17]. Based on the above discussion, it can be argued that any type of CCC increases the vulnerability of the network system. Therefore, researchers tend to investigate ‘blind rendezvous’ as a potential counter technology. Blind rendezvous is a representative technique in which each user searches for a neighbor autonomously. We will discuss some recent and acknowledged works here from the large number of rendezvous algorithms listed in Table 1.

Blind rendezvous adopts a hopping sequence that works with a list of channels [2,33], instead of a centralized node or CCC. The blind rendezvous algorithms propose a certain sequence for users to visit all the channels from the list. To generate such a hopping sequence, the blind rendezvous employs algorithmic number theory, such as slotted seeded channel hopping (SSCH) [18]. This hopping sequence requires tight time synchronization, which imposes an extra overhead. Similarly, the ring walk (RW) [19] channel hopping sequence is based on node identification (ID). The static and pre-defined nature of node ID is inadequate owing to the dynamic nature of the cognitive radio environment. Adaptive multiple rendezvous control channel (AMRCC) [20] is a channel-hopping algorithm in which users sense and rank the available channels based on the signal-to-noise ratio (SNR). However, after sensing, the users need to synchronize, which imposes an extra overhead.

**Figure 1:**
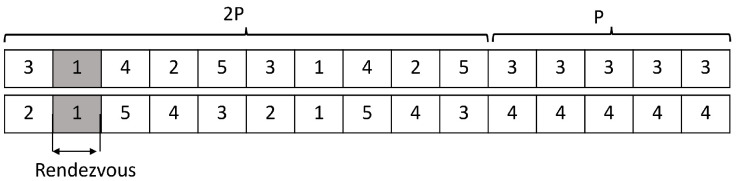
JS hopping sequence for N=4,P=5.

In [6], a generated orthogonal sequence (GOS) that requires a symmetrical model was proposed; that is, the channels in the hopping sequence are assumed to be the same. In addition, there is a possibility of unproductive utilization of channels caused by an imbalance of traffic on different channels. In modular clock (MC) and modified MC (MMC) [3], a hopping sequence is generated by a modulo operation based on some prime number and rate, which are randomly selected by each user. However, MC cannot guarantee rendezvous in the asymmetric case; therefore, an MMC is proposed. In MMC, two users should select different prime numbers to guarantee rendezvous, although they fail to assure the same prime number. In [10], jump-stay (JS) is proposed to support both synchronous and asynchronous cases. JS takes less time to rendezvous compared to the other algorithms and guarantees rendezvous within a finite time. This hopping sequence consists of two jump-pattern to hop over available channels and one stay pattern to keep staying on the same channel. We present a general JS sequence in Figure 1 as this is the basic consideration for many other approaches. Later, enhanced jump-stay (EJS) [24] modifies the JS to one jump-pattern and one stay-pattern. EJS advances the state of the art by referring to the overall smallest upper bound without any additional information.

Similar to JS, E-AHW [25] adopts an alternate hop-and-wait scheme to guarantee rendezvous. Again, this scheme is dependent on SUs’ IDs to generate a sequence that is clearly not suitable for the dynamic nature of CRNs. T-CH [26] and D-CH [26] were proposed in [26]. D-CH is ID-based; therefore, the constraint remains the same. In contrast, T-CH requires a preassigned role in successfully achieving rendezvous. In [27], the rendezvous couple channel hopping algorithm (RCCH) was proposed, but it shortfalls with the asymmetric nature of channels. RCCH increases the utilization of channels but requires a preassigned role for SUs. A time-efficient rendezvous algorithm called DSCR, which employs a disjoint set cover (DSC), was proposed in [28]. DSCR assumes that each time slot is double the length to ensure an overlap, which results in a longer TTR (i.e., time in seconds/milliseconds), although the number of slots may be reduced. The maximum diversity was achieved in [34] with a deterministic succession-based rendezvous scheme. A fast and blind rendezvous was proposed in [35] that encounters jamming attacks. P-ary m-sequence [36] proposed the first use of maximum length sequence but it was only applied to directly construct the 1-D asynchronous CH sequences and it works only for symmetric channel set. However, with uncertain channel conditions and time synchronization, there is always scope for further improvement.

In addition, rendezvous techniques with multiple radio interfaces exist. AR [9] is based on JS; however, it lacks the theoretical proof of guaranteed rendezvous. The study on AR was extended in [37]; however, both studies consider multiple interfaces to achieve rendezvous at a low rate. The authors in [38] proposed rendezvous for the homogeneous channels and those in [39] illustrated an upper bound of rendezvous, and both of these studies considered multiple-radio. The authors in [29] proposed a fair CH sequence in which all the channels have an equal probability of being used as a rendezvous channel. Once again, the role of SUs is pre-assigned, which is a major drawback. Another role-based model was proposed in [30] and where all the users follow the same symmetric role-based algorithm. However, to achieve this a strict time synchronization is required which is not considered. A matrix-based efficient rendezvous was proposed in [40], where every SU has its own local channel set. Another CH sequence was constructed using two-dimensional algebraic algorithms in homogeneous channel settings [31]. However, wireless channels are heterogeneous by nature which is a limitation of this work. Different heterogeneous conditions were suggested in [41,42], where the authors adopted multiple radios to achieve quick rendezvous. The authors in [43] present a quick rendezvous algorithm in distributed cognitive radio networks with the concepts of single radio, multi-radio, and hybrid radio. The major drawback of this study is the MTTR with a heuristic approach, which is not consistent with the probability-based rendezvous accuracy. A K-group random channel hopping (K-RCH) was proposed for both pair-wise and multi-user rendezvous [32]. The author assumed that multiple users hop on the same channel at each time so that the rendezvous time will reduce. It is mathematically correct but brings the problem of collision, disregarding all the works discussed above. The later part of the survey is motivated by this limitation.

The algorithms discussed above are strongly based on mathematical concepts that lack the consequences of collisions in real environments. Rendezvous is guaranteed when two SUs discover each other on the same channel at the same time. The assumption is that the exchange of control information is always successful. However, in a wireless environment, collisions are unavoidable because of the simultaneous transmission of control packets and data packets. Many researchers have proposed several protocols listed in Table 2 to eliminate such drawbacks; here, we discuss a few of them that exclude CCC.

Cognitive radio MAC (COMAC) was proposed in [44] based on the CSMA/CA protocol to maintain a list of unoccupied channels. Each CR user transmits channel information to the intended receiver. Based on the received information, CR users select data channels individually. However, COMAC fails to address the multichannel hidden terminal problem. Prepare-to-send (PTS) was introduced in [45] along with classic ready-to-send(RTS)/clear-to-send(CTS) as a new handshake procedure, namely CR-CSMA/CA. Through PTS, all CR users can be notified about the current time reservation for spectrum sensing. This new control packet can cause overhead, and the users who overhear the PTS can update their network allocation value (NAV) accordingly. To minimize collision, a concurrent access MAC (CA-MAC) protocol was proposed in [46], which maintains two channel lists: (i) sorted channel list (SCL) and (ii) common channel list (CCL). The drawback of CA-MAC is that it maintains a global SCL that requires frequent exchange of channel information.

CR-RDV was proposed in [49], which modifies the control packets (i.e., RTS/CTS) to work with the asynchronous channel list. It revisits the traditional back-off procedure to conserve the PU transmission. The modified RTS/CTS packet carries the available channel list (ACL) and channel occupancy list (COL). Therefore, the control-information overhead increases proportionally with the increase in a number of channels. In [47], a MAC protocol based on CSMA/CA was proposed to consider the available channel status, congestion of users, and collisions on channels. They try to handle collisions in the rendezvous process between PUs and SUs using queuing theory and a control scheme. In [13], a slot-asynchronous MAC based on the RTS/CTS handshake mechanism was proposed. This mechanism improves the handshake performance during the channel hopping process by mitigating the effect of the asynchronous time slot but faces an additional handshake failure problem, which is harmful to the network throughput. In [48], the authors employed correlation-based signal detection to propose a cooperative control feedback scheme that avoids back-off misbehavior. The key idea of this scheme is to transmit false collision notifications from neighbors to differentiate the intended receiver failure. This approach requires symbol-level synchronization between the users.

It should be noted that there are some differences between our study and the above-mentioned studies. First, we propose a CH scheme with a guaranteed short TTR, which is not guaranteed by the other CH schemes. Second, when SUs hop on the same channel, the proposed MAC ensures that the opportunity is not wasted. We combine these two in this study because a CH scheme cannot avoid collisions, and similarly, MAC can ensure a small TTR. CH and MAC complement each other to achieve successful rendezvous.

## 3. System Model

We consider a CRN where a finite number of SUs and PUs are distributed in a single contention domain. The potential spectrum is divided into *N* non-overlapping orthogonal channels, indexed as 1,2,⋯,N, and the channel indices are well known to all SUs. Each SU with its equipped half-duplex radio can switch to any channel; however, it can work only on one channel at a time. We assume a self-organizing network in which SUs can communicate with each other if they are within the transmission range. In the present model, PUs are the authorized owners of spectrum bands and access the channel in a synchronous time-slotted manner. All the channels have the same bandwidth and are recognized by the central frequency. Each channel holds an equal space of bandwidth from the next adjacent channel, as in most wireless systems, such as IEEE 802.11 [50].

A channel is available to SU if there is no interference during transmission. With the help of an appropriate sensing model [51], SUs can find channels from available *N* channels before the rendezvous process. Here, we consider a symmetric model, that is, all SUs have an equal number of channels if they are in the same vicinity. In contrast, a PU can randomly select a channel to carry out data transmission in a slot-by-slot manner. Therefore, multiple PUs can select the same channel simultaneously, which leads to a crisis in which a channel is unavailable for rendezvous at any time.

**Figure 2:**
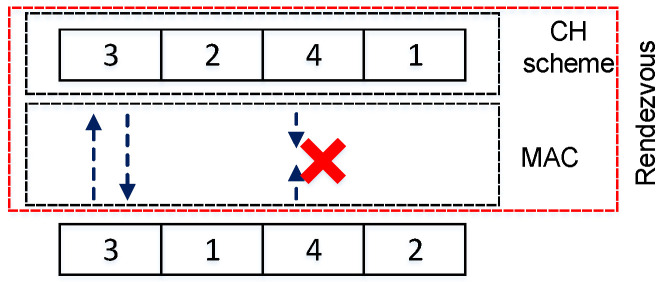
Structure of rendezvous.

We considered an asynchronous network model, that is, there is no global time synchronization among SUs. However, the duration of a slot should be long enough to complete the rendezvous process. For analytical simplicity, we assumed that the clock difference between two SUs is a random integer [52], the number of mini-slots [52]. In this study, we considered reliable active scanning (RAS), which is simple and efficient for detecting loss of probe request and fast retransmit or hop on the next channel. However, successful transmission of the probe request is unpredictable owing to some collision probability and the lack of acknowledgment. In this active scanning, an SU broadcasts a probe request and expects to receive a probe response from any neighboring SU. Figure 2 depicts the basic structure of the rendezvous process considered in this study. Two users can hop on different channels; however, when both hop on the same channel, there should be a successful exchange of messages which is indicated by the arrows in Figure 2. In an worst-case scenario, users on the same channel could experience collision as shown on channel 4 in Figure 2 with the cross sign.

For the blind rendezvous process, we particularly selected the probe request and response messages. Most studies on rendezvous have considered RTS/CTS for user discovery, which is unrealistic. The RTS packets are unicast; therefore, a specific destination address must be provided in the RTS packet. In the legacy 802.11 system, RTS/CTS are used for channel reservation when the destination AP address is known. In contrast, in a rendezvous process, the user has no information about the other users in the network. We consider rendezvous as an initialization process in which a user tries to connect with any other user in the network. Hence, there is no specific destination address that is known to the user.

### Rendezvous Mechanism

**Figure 3:**
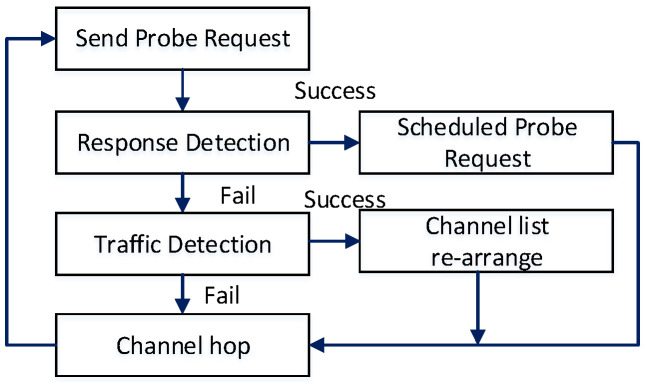
Process to achieve rendezvous.

Channel availability is flexible during channel hopping, and SUs must detect if the channel is free from the incumbent or any other SUs. We integrated 802.11-based active scanning with the channel-hopping scheme. According to the active scanning method, a station actively broadcasts a probe request frame on the current channel and expects to receive a probe response from the access points (APs) [53]. Similarly, in the cognitive network structure for rendezvous, the response frame is from the SUs who have successfully received the probe request. On some channels, there are no SUs to receive the probe request, while others may have more than one SU. After sending the probe request, there are two steps: response detection and traffic detection, according to Figure 3. The response time is a small duration reserved exclusively for a probe response frame. If an SU does not detect any probe response during this period, the probe request is not delivered properly. Upon successful response detection, the rendezvous process will be complete, and the user will schedule for the next rendezvous attempt. The traffic detection occurs after the response time when the SU fails to detect any transmission. If the SU successfully detects any packet or collision during the traffic detection time, it determines that this is an active channel, and the probe request is lost. Based on this, the SNR values of the channels will be rearranged for the next 2P.

The unit time at which each SU visits a channel is defined as a slot. In this study, the hopping sequence in Section 4 works in the unit of slots, and the MAC protocol in Section 5 works in the unit of mini-slots within a slot. In other words, the TTR of the hopping sequence is counted as the number of slots, and the TTR of the MAC protocol is counted as the number of mini-slots. For example, probe requests and response messages in our proposed MAC protocol are sent on a selected mini-slot within the visited slot. In this study, the hopping sequence works for asynchronous slots, which means that the starting time of a slot may be different for users.

## 4. Hopping Sequence Generator

We now present the proposed hopping sequence, V-HS, for symmetric and asymmetric models. Any two users individually generate their own sequence following the same set of rules. In the present model, the entire set of non-overlapping orthogonal channels is denoted by $C=\{c_{1},c_{2}\ldots ,c_{N}\}$. Let $C_{n}\subseteq C$ denote the set of available channels for user n,n={1,2,…}. The number of commonly available channels between the users is denoted by *G*, that is, $G=C_{1}\cap C_{2}$. In the symmetric model, both users have the same available channel, that is, $C_{1}=C_{2}$. In contrast, an asymmetric model presents both users with different sets of available channels. If there is no channel common between these two sets, rendezvous is impossible. Therefore, a feasible solution assumes that at least one channel is common between these two sets, that is, G≠{}.

Although users have *N* channels, they require the selection of another parameter related to *N*. Let *P* be the smallest prime number greater than *N*. Each round lasts for approximately 2P time slots. For every 2P time slot, users generate a sequence based on predefined parameters. Theorems 1 and 2 explain how the sequence is generated consecutively for both the symmetric and asymmetric models.

### 4.1. Channel Hopping Sequence Generator

**Theorem** **1** (Channel hopping sequence for symmetric model)**.** *Let N be the number of channels and P be the smallest prime number greater than N. Let s(t) be the hopping sequence of period* 2*P defined as*

$$s\left(t\right)=\left\{\begin{array}{cc} ((rt/2\;+\;i)\;mod\;P)+1 & for\;t\;\equiv \;0\;mod\;2\\ r & for\;t\;\equiv \;1\;mod\;2 \end{array}\right.$$

*where r is the index of the best channel, which is naturally a position integer indicating a channel number such that* 1 ≤ *r < P and the greatest common divisor between r and P is, gcd(r,P)* = 1. *i is the time difference between users given as a random positive integer less than P. Then, two users will appear on the same channel within* 2*P time slots.*

**Figure 4:**
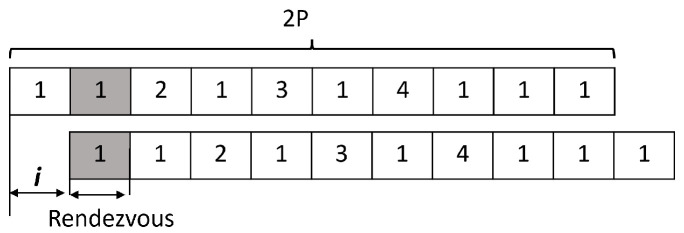
V-HS for *N* = 4, *P* = 5, *r* = 1, *i* = 1.

Suppose that the channel indices are [1 2 3 4], then N=4 and P=5. When s(t) generates a number that exceeds the indices, s(t)>N, both users replace each exceeded number with the same index. For instance, f(t)=5 and both users change s(t) to the same number *a*, such as 1≤a≤4. Figure 4 presents a generic view of our proposed V-HS where all the channels are visited within 2P. Here, r=1 indicates that the channel in the index 1 (i.e., channel 1) is with the best SNR value. From a careful observation, it will be clear that for any value of *i* (i.e., *i* <2P), there will be a rendezvous.

To prove the above theorem, we consider the following lemmas. Here, s(t) is the channel-hopping sequence of a symmetric system.

**Lemma** **1** (Lin, Liu, Chu, and Leung [10])**.** *Given a positive integer n, if* r∈[1,n] *is relatively prime to n, i.e., the common factor between them is* 1*, then for any* t∈[0,n] the sequence s(t)=<t%(n+1),(t+r)%(n+1),…,(x+(n−1)r)%(n+1) *is a permutation of* <1,2,…,n>.

**Lemma** **2** (Lin, Liu, Chu, and Leung [10])**.** *Given a prime P, if $r_{1}$ and $r_{2}$ are two different numbers in (1,P), then for any $x_{1}\in \left[1,P\right]$ and $x_{2}\in \left[1,P\right]$, there must be an integer k∈[1,P] such that $\left(x_{1}\;+\;kr_{1}\right)\%P=\left(x_{2}\;+\;kr_{2}\right)\%P$.*

**Proof**  The proof is given for three cases.

Case (1) The time difference *i* between two users is odd. This is because *r* is a position integer less than or equal to *P* and assigned to all odd time slots that meet the even time slot of other users. There should be time slots for the other users with channel index *r*. Therefore, a rendezvous occurs within 2P time slots.

Case (2) The time difference between two users is even, and *r* of the two users are different. According to the results of Lemmas 1 and 2, there should be a rendezvous in an even time slot within 2P time slots (*P* even time slots).

Case (3) The time difference between two users is even, and *r*’s of the two users are the same. Because every odd time slot has a channel index *r*, if two users have the same *r*, then a rendezvous occurs in every odd time slot. Using the channel-hopping sequence for the symmetric model in Theorem 1, we can generate a channel-hopping sequence for the asymmetric model, as in the following theorem. □

**Theorem** **2** (Channel hopping sequence for the asymmetric system)**.** *Let *N* be the number of channels in the communication environment, *G* be the number of common channels between two users, and *P* be the smallest prime number greater than *N*. Let $r_{1}$ and $r_{2}$ be the indices of the best channel of users 1 and 2, such that $1\leq r_{1},r_{2}<P$. Let the channel-hopping sequence $q_{i}\left(t\right)$ for user i(i=1,2) be defined as follows:*

$$q_{i}\left(t\right)=\left\{\begin{array}{cc} (\left(r_{i}t/2+\left\lfloor \frac{t}{4P}\right\rfloor \right)\;\mathrm{mod}\;P)+1, & \mathrm{for}\;t\equiv 0\;\mathrm{mod}\;2\\ (\left(r_{i}+\left\lfloor \frac{t}{4P}\right\rfloor \right)\;\mathrm{mod}\;P)+1, & \mathrm{for}\;t\equiv 1\;\mathrm{mod}\;2 \end{array}\right.$$

Then a rendezvous occurs within 4P(P−G+1) time slots. When $q_{i}\left(t\right)$ generates a number that exceeds the indices, that is, $q_{i}\left(t\right)>G$, the number is replaced with an index of $r_{i},\left(i=1,2\right)$. For instance, if $q_{i}\left(t\right)=5$, it will be changed to $q_{i}\left(t\right)=2$ if $r_{i}=2$.

**Proof.**  For an integer *k* such that 1≤k<P, let $a_{i}^{k}\left(t\right)$ is defined as follows.

$$a_{i}^{k}\left(t\right)=\left\{\begin{array}{cc} (\left(r_{i}t/2+k\right)\;\mathrm{mod}\;P)+1, & \mathrm{for}\;t\equiv 0\;\mathrm{mod}\;2\\ (\left(r_{i}+k\right)\;\mathrm{mod}\;P)+1, & \mathrm{for}\;t\equiv 1\;\mathrm{mod}\;2. \end{array}\right.$$

□

**Figure 5:**
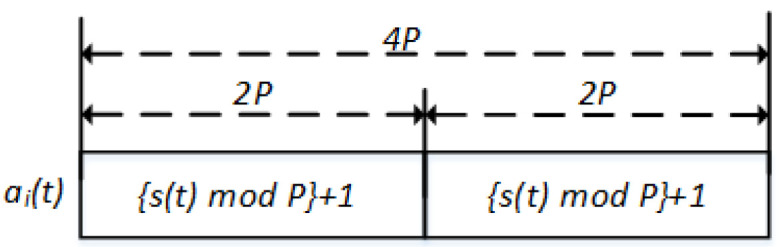
Relation between $a_{i}^{k}\left(t\right)$ and s(t).

In the above equation, $a_{i}^{k}\left(t\right)$ has the repeated form of the sequence in Theorem 1. Using $a_{i}^{k}\left(t\right)$, $q_{i}\left(t\right)$ can be written as follows:

$$q_{i}\left(t\right)=a_{i}^{k}\left(t\right),\mathrm{for}\;k=1,2,\cdots ,P-1,$$

where $a_{i}^{k}\left(t\right)$ can be rewritten using s(t) in Theorem 1, as shown in Figure 5.

**Figure 6:**
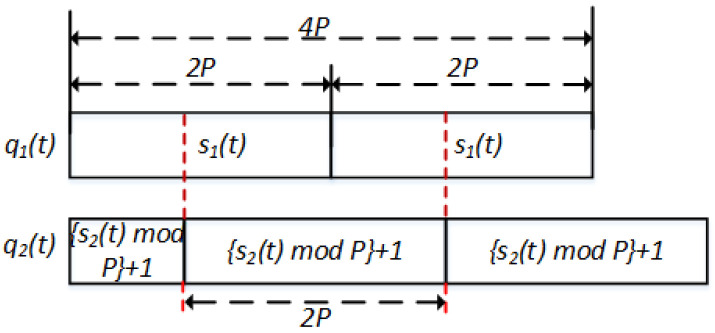
Channel hopping sequence in first 4P time slots.

Let $i_{1}$ and $i_{2}$ be the initial times for users 1 and 2, respectively. Without loss of generality, it can be stated that $i_{1}\leq i_{2}$. Subsequently, we can define $i_{1}^{\prime }=0$ and $i_{1}^{\prime }=i_{2}-i_{1}$, which can be seen as the initial time for users 1 and 2, respectively. Figure 6 depicts the sequence $q_{1}\left(t\right)$ and $q_{2}\left(t\right)$ with a time difference $i_{2}^{\prime }$.

Let us consider the first 4P slots of $q_{i}\left(t\right)$ with the existence of the time difference $i_{2}^{\prime }$. In the first 4P time slots, $a_{1}^{0}\left(t\right)$ satisfies $a_{2}^{k}\left(t\right)$ with a time difference of $i_{2}^{\prime }$. Without loss of generality, we can set $0\leq i_{2}^{\prime }<4P$. As shown in Figure 6, there always exist 2P time slots that contain $s_{1}\left(t\right)$ and $s_{2}\left(t\right)+k\;\mathrm{mod}\;P$.

From the result of Theorem 1, there must be the same symbol in 2P time slots. Because $a_{i}^{k}\left(t\right)\equiv a_{i}^{0}+k\;\mathrm{mod}\;P$, if there is a time slot with the same symbol at $a_{1}^{0}\left(t\right)$ and $a_{2}^{k}\left(t\right)$, then $a_{1}^{m}\left(t\right)$ and $a_{2}^{k+m}\left(t\right)$ also have the same symbol at the same time slot as the value increases by *m*.

Therefore, users 1 and 2 show all channels in the environment simultaneously within $4P^{2}$ time slots. Because the two users have *G* common channels, a rendezvous occurs within 4P(P−G+1) time slots, using $q_{i}\left(t\right)$ as the channel-hopping sequence.

### 4.2. Expected TTR of Symmetric Model

Based on Theorem 1, the following corollary, which provides an expected TTR of V-HS in the symmetric model, is obtained.

**Corollary** **1.** *The expected TTR of the system using the V-HS in Theorem 1 is*$\left(2P^{2}-P+1\right)/\left(2P\right)$.

**Proof.**  To calculate the expected TTR, the following three cases need to be considered:

Case (1) $r_{1}=r_{2}$ with even time difference.

It is clear that the probability of occurrence of this case is 1/(2P). When $r_{1}=r_{2}$, the two users have the same symbol in even time slots. Therefore, a rendezvous occurs at time slot 0. Hence, the expected TTR is 1.

Case (2) $r_{1}\neq r_{2}$ with even time difference.

It is clear that the probability of occurrence of this case is (P−1)/(2P). When $r_{1}\neq r_{2}$, the two users have different symbols in even time slots. Therefore, a rendezvous cannot occur in even time slots. For odd time slots, the probability of rendezvous is equal for every time slot. Hence, the expected TTR is

(1) $$\frac{1}{P}\sum_{i=1}^{P}\left(2i-1\right)=\frac{P(P+1)}{P}-1=P.$$

Case (3) Odd time difference.

It is clear that the probability of occurrence of this case is 1/2. In this case, one user’s odd time slot always meets the other user’s even time slot. Therefore, a rendezvous occurs at the time slot where user 1’s odd time slot has the value $r_{2}$, or user 2’s odd time slot has the value $r_{1}$. In this case, the probability of rendezvous is equal for every time slot. Therefore, the expected TTR in this case is *P*.

From the results of Cases (1)–(3), the expected TTR can be calculated as

(2) $$\begin{array}{cc} E[{\mathrm{TTR}}_{\mathrm{sym}}^{s}]\; & =\frac{1}{2P}\times 1+\frac{P-1}{2P}\times P+\frac{1}{2}\times P\\ \; & =\frac{1}{2P}+\frac{P(P-1)}{2P}+\frac{P^{2}}{2P}\\ \; & =\frac{2P^{2}-P+1}{2P}. \end{array}$$

□

### 4.3. Expected TTR for Asymmetric Model

Based on Theorem 2, the following theorem, which provides an expected TTR in the asymmetric mode, can be obtained.

**Theorem** **3.** *The expected TTR of the system using V-HS in Theorem 2 is calculated as*

(3) $$E[{\mathrm{TTR}}_{\mathrm{asym}}^{s}]=\sum_{k=1}^{P}\left\{4P\left(k-1\right)+\frac{4P^{2}-P+1}{2P}\right\}\left\{G{\left(P-G\right)}^{k-1}\prod_{j=0}^{k-1}\frac{1}{P-j}\right\}.$$

Before beginning to prove the above theorem, we should see the following lemma.

**Lemma** **3.** *Let N be the number of channels in the system, P be the smallest prime number greater than N, and G be the number of common channels between two users who want to rendezvous. Then, using the channel-hopping sequence of Theorem 2, the probability that a rendezvous occurs at* $a_{i}^{k}\left(t\right)$ *-k-th component sequence is calculated as*

(4) $$G{\left(P-G\right)}^{k-1}\prod_{j=0}^{k-1}\frac{1}{P-j}.$$

**Proof.**  As the number of common channels is *G*, it is clear that the probability that a rendezvous occurs at the first component sequence is G/P. □

Now, considering the case in which a rendezvous occurs at the second component sequence. This means that a rendezvous does not occur at the first component sequence. Therefore, the channel index that is located at the same time slot for both users’ channel-hopping sequences is not on the list of common channels. Therefore, this channel needs to be removed to calculate the probability of rendezvous at the second component sequence. With this process, we can calculate the probability that a rendezvous does not occur at the first component sequence is (P−G)/P, and that a rendezvous occurs at the second component sequence is G/(P−1).

Using a process similar to that of the second component sequence, the probability of rendezvous at the *k*-th component sequence can be calculated as given in Equation (4).

**Proof of Theorem** **3.** According to Theorem 2, the channel-hopping sequence $q_{i}\left(t\right)$ consists of the component sequence $a_{i}^{k}\left(t\right),\;k=0,\cdots ,P-1$, and $a_{i}^{k}\left(t\right)$ is a duplication of $s_{i}\left(t\right)$ in Theorem 1. As shown in Figure 2, the average time-frame slot until the rendezvous starts in $s_{i}\left(t\right)$ is *P*. Using the result of Corollary 1, if $a_{i}^{k}\left(t\right)$ has a common channel between both users, the average TTR in $a_{i}^{k}\left(t\right)$ is $\left(2P^{2}-P+1\right)/\left(2P\right)+P=\left(4P^{2}-P+1\right)/\left(2P\right)$. Because $a_{i}^{k}\left(t\right)$ is the *k*-th component sequence of $q_{i}\left(t\right)$, the actual expected TTR for $a_{i}^{k}\left(t\right)$ is $4P\left(k-1\right)+\left(4P^{2}-P+1\right)/\left(2P\right)$. From the result of Lemma 3, the probability of rendezvous at $a_{i}^{k}\left(t\right)$ is $G{\left(P-G\right)}^{k-1}\prod_{j=0}^{k-1}1/\left(P-j\right)$. Therefore, the expected TTR for the asymmetric model using the channel-hopping sequence in Theorem 2 can be calculated using Equation (3). □

## 5. Design of the Rendezvous MAC Protocol

Although V-HS has been proven to show better TTR, a rendezvous in reality will be achieved by a message exchange between two users. We now describe the proposed rendezvous MAC protocol, ReMAC, that can be integrated with V-HS as well as any other channel-hopping scheme.

### 5.1. Description of the Protocol

#### 5.1.1. Rendezvous Attempt Using Probe Request and Response

**Figure 7:**
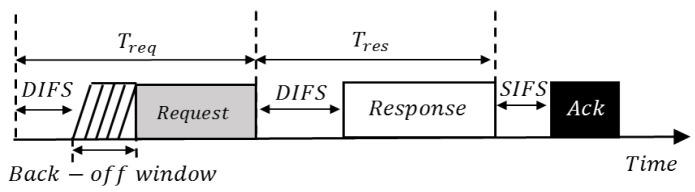
Basic structure of exchanging a probe request and response.

Two SUs achieve a rendezvous with each other by exchanging some messages. As stated earlier, we propose to use probe request and probe response packets for such a purpose, which is similarly used for scanning in the IEEE 802.11 systems. We define a rendezvous attempt (RA) as the duration to exchange a probe request and response. Figure 7 depicts the basic structure of an RA in which a pair of probe requests and responses are exchanged. Here, $T_{req}$ and $T_{res}$ are probe request and response time, respectively. During the DCF interface space (DIFS), a user will listen to a channel and if found busy, it will differ the transmission time for the probe request. Short interface space (SIFS) is the amount of time required to proceed with the received request and after this period, the user will send a response.

However, these probe messages are exposed to collisions or interference from PUs or other SUs. In particular, when too many SUs try to send probe messages for rendezvousing, collisions are inevitable. Collisions during rendezvous can be classified into the following types: (i) Collisions between probe requests/responses (from SUs) and data packets (from PUs), and (ii) collisions between control packets. CSMA/CA can resolve the first type of collision by checking the busy-medium conditions. However, the collisions between probe requests and responses are unavoidable in a protective way because of the property of blind rendezvous. We design a protocol that considers such collision possibilities and retransmissions.

#### 5.1.2. Analysis of Collision

Based on the classification of control packets, the collision during a rendezvous process can be sorted into three types: (i) Collision between probe requests, (ii) collision between probe response, and (iii) collision between probe requests and responses.

**Figure 8:**
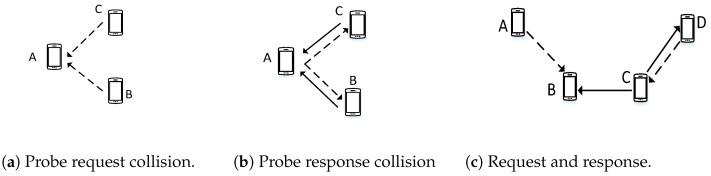
Different cases of collisions.

In Figure 8, the dashed line represents the probe request, and the solid line represents the probe-response message. The first type of collision occurs because of the asynchronous nature of SUs. If a channel is free from a PU, any SU can attempt rendezvous on that channel; therefore, probe request collisions are obvious. In Figure 8b, SU *A* transmits a request and receives a collision owing to the simultaneous transmission of the probe response from SUs *B* and *C*. Compared to classical wireless networks, the number of probe response messages is higher in CRNs owing to the large number of SUs. The hidden terminal problem causes probe response and probe request collisions, as shown in Figure 8c.

These collisions between probe requests and responses cannot be avoided entirely in a proactive way because of the property of blind rendezvous. However, an MAC that can reduce the impact of collisions during the rendezvous process can be designed.

#### 5.1.3. Partial Probe Request/Response Analysis

In CRNs, all SUs attempt rendezvous at random times. Therefore, another reason for rendezvous failure is the partial recipient of the probe request/response. In asynchronous CRNs, SUs can receive a part of a message, which wastes many potential opportunities.

**Figure 9:**
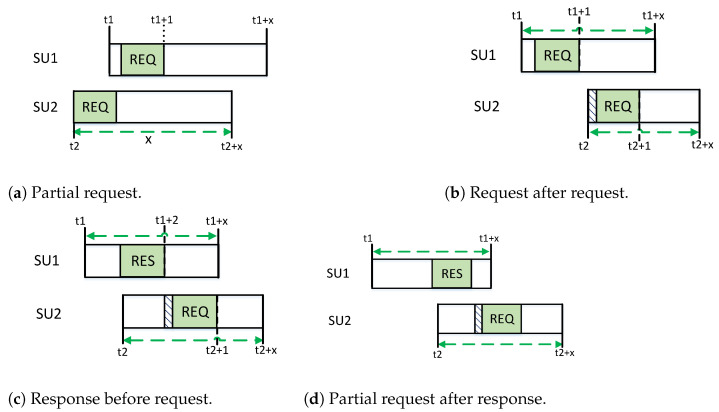
Different cases of partial probe request and response.

As illustrated in Figure 9, the two SUs attempt to rendezvous on the same channel but fail because of their asynchronous nature. Suppose that SU1 and SU2 hop on the same channel at times $t_{1}$ and $t_{2}$, respectively. Suppose that the normalized time for a probe request/response is 1. Let us assume that the length of a time slot is *x* and therefore, to complete at least on a pair of probe requests and response *x* should be longer than 2. To guarantee an overlapping time between the two SUs on a channel, the constraint $|t_{1}-t_{2}|\leq x$ is imposed. Based on Figure 9, there are a few conditions to be satisfied for a successful rendezvous.

According to Figure 9a, if $t_{1}>t_{2}$ (SU1 hops later than SU2), SU1 receives a partial part of SU2’s request and sends a probe request to SU2. Therefore, the leaving time of SU2 should be greater than *x*, that is, $t_{2}+x\geq t_{1}+2$. Similarly, in Figure 9b, where $t_{2}>t_{1}$, the leaving time of SU1 should be $t_{1}+x\geq t_{2}+2$. In Figure 9c, $t_{2}>t_{1}$ (SU2 hops later than SU1) and overhears a probe response from SU1. This manifests the presence of an SU; hence, SU2 sends a request message to SU1. Therefore, the leaving time of SU1 in both Figure 9c, and Figure 9d should be $t_{1}+x\geq t_{2}+1$. The length of *x* can vary based on the collision and partial probe request/response. The optimal value for *x* is outside the scope of this study; therefore, we consider that *x* does not exceed the size for basic active scanning in 802.11 [53]. This design consideration will enhance the probability of rendezvous compared to that of traditional designs.

#### 5.1.4. ReMAC Protocol

**Figure 10:**
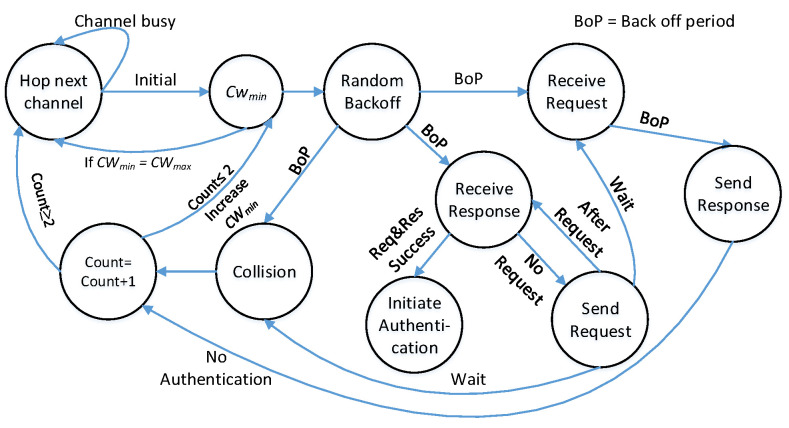
State diagram of ReMAC protocol.

The state-transition diagram in Figure 10 presents the working principle of the proposed ReMAC protocol. Let us consider two SUs *A* and *B*, who are about to attempt rendezvous. *A* and *B* hop on an initial channel based on V-CH. The state ‘hop next channel‘ follows the proposed V-CH to decide the next channel for the rendezvous attempt. Initially, both *A* and *B* hop on a channel and check whether or not the channel is busy. If the channel is busy, both SUs hop on the next channel according to V-CH. If the channel is found empty, the SUs can determine a random backoff time with its current $CW_{min}$. During this backoff period, SUs can receive a request or response, or even sense a collision. Suppose that *B* receives a request on the channel from *A*, *B* will send a response message. In the best case, *A* will receive the response properly, and a rendezvous will be achieved. However, the following three possible scenarios need to be considered: (i) *B* receives a collision or response before receiving or sending a request, (ii) *B* hops later and sends a request to *A*, and (iii) *A* and *B* send a request at the same time.

In ReMAC, for (i) *B* will send a probe request. *B* has not sent any probe request until now; therefore, with the initial back-off time, *B* attempts the first request if it receives a response. However, for a collision, *B* will increase the count and $CW_{min}$ to generate a new backoff. In case (ii) *A* will send a response to the request from *B*. If *B* receives the response properly, a rendezvous will be achieved, and *B* will initiate the authentication process. When sending a request/response, the sender cannot listen to the channel; therefore, for case (iii) *A* and *B* will not sense any collision or receive any response. After the first rendezvous attempt, *A* and *B* double their back-off time for any of the following three conditions: (i) sense collision, (ii) no authentication, and (iii) receive no response. With this new back-off, both *A* and *B* will attempt rendezvous for the last time following the explained procedure. However, failing to rendezvous during this attempt will cause them to hop on the next channel following the V-CH.

#### 5.1.5. Collision Resolution Using CSMA/CA

To resolve the possibility of collision, Re-MAC is integrated with the CSMA/CA mechanism of the IEEE 802.11 protocol that supports random access and collision resolution with a back-off window. When an SU hops to a channel and if the channel is sensed idle for DIFS, the SU will select a value randomly between [$0,CW_{min}-1$] to set its back-off counter. During the back-off counting-down process, the back-off counter is decremented by one, and when it is 0, the SU broadcasts a probe request. Upon receiving a probe request, the receiver SU follows the same process to reply with a probe response. Finally, the transmitter SU sends an ACK for confirmation after receiving a probe response because there is a possibility of collision of probe responses from multiple SUs or any PU. Here, $T_{req}$ and $T_{res}$ represent the time consumed to send a probe request and to receive a probe response, respectively. Note that setting an accurate $CW_{min}$ is not trivial, because if $CW_{min}$ is too large, the channel-access delay will increase [54], and if $CW_{min}$ is too small, the collision probability will increase (For equivalence with the IEEE 802.11 systems, where $CW_{min}=15$, $CW_{min}$ in our system model can be smaller because the randomness increases over several channels as well as over the contention window. In the simulation, we will study the effect of $CW_{min}$ on the performance).

#### 5.1.6. Multiple Rendezvous Opportunities in a Slot

**Figure 11:**
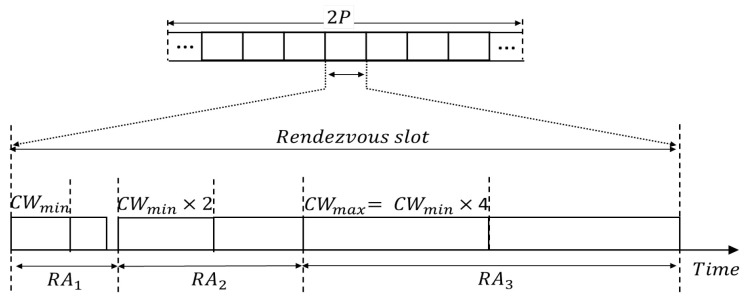
Multiple rendezvous opportunities in a slot in the case of three RAs.

Once a probe request or response fails because of collision or any other reason, the SUs may try a rendezvous attempt as long as the remaining mini-slots in the slot are sufficient for the second RA. In most rendezvous studies, multiple rendezvous opportunities in one slot have not been considered. Because of multiple RAs, the chances of rendezvous failure due to collision reduce and the rendezvous opportunity increases. Figure 11 shows an example of three RAs. Let $RA_{max}$ be the maximum number of retransmissions in a slot (In IEEE 802.11, the maximum retransmissions is 7 by default and it is 4 for RTS/CTS [55]. Figure 11 presents an example of three retransmissions, that is, $RA_{max}=3$. This number is also a design parameter that fits within the length of a slot).

**Figure 12:**
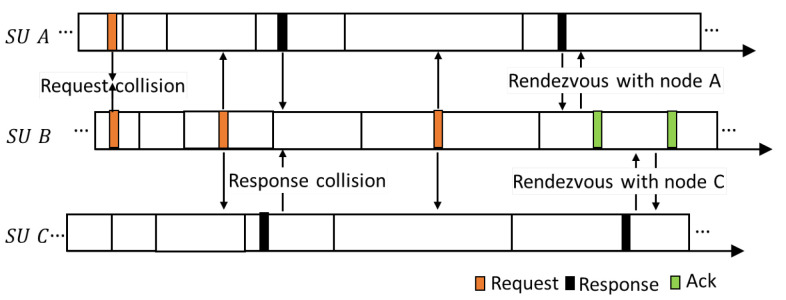
An example of collision resolution of probe messages.

Let $T_{req,i}$ and $T_{res,i}$ represent the time to send a probe request and response, respectively, at *i*-th RA, $RA_{i}$; then, they are expressed as [55]

$$T_{req,i}=DIFS+rand\left(0,CW_{i}-1\right),$$

$$T_{res,i}=DIFS+slotTime\times CW_{i},$$

where $CW_{i}$ is the contention-window size at $RA_{i}$ and $CW_{i}=2^{i-1}\times CW_{min}$ and $CW_{1}=CW_{min}$, following IEEE 802.11. These parameters are reset whenever a new slot starts because SUs switch to another channel per slot, and thus the channel status changes at every slot. When an SU does not receive any response during $T_{res,i}$, $CW_{i}$ is doubled and the probe request is re-transmitted. Simultaneously, the time $T_{res,i}$ for receiving probe response is also doubled. Both $T_{req,i}$ and $T_{res,i}$ are generated with a doubled contention window because it is impossible to determine whether the probe request fails or the probe response fails. This procedure is depicted in Figure 11. If an SU cannot succeed in any rendezvous by transmitting the probe request $RA_{max}$ times, rendezvous fails in that slot; therefore, rendezvous may not be achieved in the MAC layer, although rendezvous is declared from the jumping sequence. Figure 12 depicts the collision of probe requests between SU *A* and SU *B*. According to legacy-channel hopping schemes, in such a condition, SUs fail to rendezvous and hop to the next channel, which is a waste of rendezvous opportunity. In contrast, in the proposed scheme, the SUs attempt rendezvous for the second time (i.e., in the next RA) with an increased back-off window as long as the remaining mini-slots in the slot are sufficient for the second RA. In Figure 12, it can be observed that the probe responses from SU *A* and *C* also collide. Accordingly, the users attempt rendezvous for the third time with an increased back-off window. Finally, SU *B* rendezvouses with SU *A* and *C*.

### 5.2. Expected TTR in MAC Layer

Using the designed rendezvous MAC protocol, we can obtain the expected TTR in units of mini-slots from the perspective of the MAC layer. For simplicity, we consider the collisions of probe messages only. A channel becomes unavailable if it is occupied during or before a rendezvous. The IEEE 802.11 systems are considered slotted (equivalently mini-slotted in our system), and the counting-down processes of all the SUs are homogeneous [56]. Therefore, the transmission probability τ of an SU can be obtained using the following Bianchi model [56]:

(5) $$\tau =\frac{2(1-P_{c})}{\left(1-2P_{c}\right)\left(W+1\right)+WP_{c}{\left(1-{\left(2P_{c}\right)}^{m}\right)}^{\prime }}$$

where $P_{c}$ denotes the probability of collision and depends on the number of users attempting to transmit on the same channel. Here, $P_{c}$ can be expressed as $P_{c}=1-{\left(1-\tau \right)}^{M}$, where *M* is the number of other users. Therefore, the probability of a successful probe request can be calculated as $P_{req}^{\circ }=1-P_{c}$. Similarly, the probability of a successful probe response can be calculated as $P_{res}^{\circ }=1-P_{c}$ for *M* other nodes who are trying to respond to the successful reception of the probe request.

Let $P_{req}\left(k\right)$ and $P_{res}\left(k\right)$ denote the probability of successful probe request and response, respectively, at the *k*-th RA in a slot. Then, $P_{req}\left(k\right)$ is given under the condition that either a failure of the previous probe request or a failure of the previous probe response at every RA for 1,…,k−1. In addition, $P_{res}\left(k\right)$ is given under the condition that the probe request at the *k*-th RA is successful. Therefore, for k≥2, they are expressed as

(6) $$\begin{array}{c} P_{req}\left(k\right)\;\;=\;\;P_{req}^{\circ }\;\;\prod_{m=1}^{k-1}\;\;\left(1\;\;-\;\;P_{req}\left(m\right)\;\;+\;\;P_{req}\left(m\right)\left(1\;\;-\;\;P_{res}\left(m\right)\right)\right), \end{array}$$

(7) $$P_{res}\left(k\right)=P_{res}^{\circ }\times P_{req}\left(k\right).$$

Note that $P_{req}\left(1\right)=P_{req}^{\circ }$ and $P_{res}\left(1\right)=P_{res}^{\circ }$.

The expected TTR under a condition that rendezvous occurs at the *j*-th slot with probability $P_{R,j}$ is calculated as follows:

(8) $$\begin{array}{c} E\left[TTR_{MAC}|P_{R,j}\right]=\sum_{l=1}^{j-1}T_{slot}+\sum_{k=1}^{RA_{max}}P_{req}\left(k\right)P_{res}\left(k\right)\\ \cdot \left\{\sum_{m=1}^{k}T_{req,m}+\sum_{n=1}^{k}T_{res,n}+SIFS+ACK\right\}, \end{array}$$

where $T_{slot}$ denoted the length of a slot, and $\sum_{l=1}^{j-1}T_{slot}\equiv 0$ for j=1. By using Equation (6), the probability that rendezvous fails at the *j*-th slot, $P_{F,j}$, is calculated as

(9) $$P_{F,j}=1-P_{R,j}=1-P_{req}\left(\psi \right)+P_{req}\left(\psi \right)\left(1-P_{res}\left(\psi \right)\right),$$

where $\psi =RA_{max}$.

Finally, we obtain $E[TTR_{MAC}]$ as follows:

(10) $$E\left[TTR_{MAC}\right]=\sum_{j=1}^{\infty }E\left[TTR_{MAC}|P_{R,j}\right]\times P_{R,j},$$

which can be also expressed in terms of Equations (8) and (9).

## 6. Performance Evaluation

We conducted an event-driven simulation to evaluate this study using MATLAB. In the simulation, the topology of a single-hop CRN was set up by randomly distributing the PUs and SUs in an area of approximately 100 m × 100 m. The transmission range of both an SU and a PU was set to 250 m. It was assumed that each PU randomly chooses a channel if they have any packet to deliver. It was assumed that the PU activity follows a Poisson distribution, and the receiver is also randomly chosen. The probability of channel availability was set to 50% by adjusting the ON-OFF channel-availability model throughout our simulation. If two users are in the vicinity, there is a channel correction between them. For each channel, we have generated a noise level that is determined by path loss and some deviation. This deviation follows a log-normal distribution with 6 db variance like shadowing. Eventually, the noise level varies between −120 dBm and −95 dBm for users. We assume that for each iteration, the noise level of a channel does not change. Each result was averaged by approximately 1000 runs of simulation with identical parameters. Mobility was not considered in this study. The performance was measured in terms of TTR in units of slots for the hopping sequence and in units of milliseconds for the MAC protocol.

The evaluation of this study comprised two parts: first, the performance evaluation of V-HS, which was compared with those of MMC, JS, and AR for symmetric and asymmetric models and second, the performance evaluation of ReMAC measured with CAMAC and CR-RDV. CR-RDV is comprised of phases: (1) rendezvous phase and (2) data exchange phase. For a fair comparison, we considered the rendezvous phase to collate with. Although MMC, JS, and AR handle a hopping pattern without MAC, we imposed our MAC on them by setting general parameters to evaluate the performance. Table 3 lists the parameters used for the simulation.

**Figure 13:**
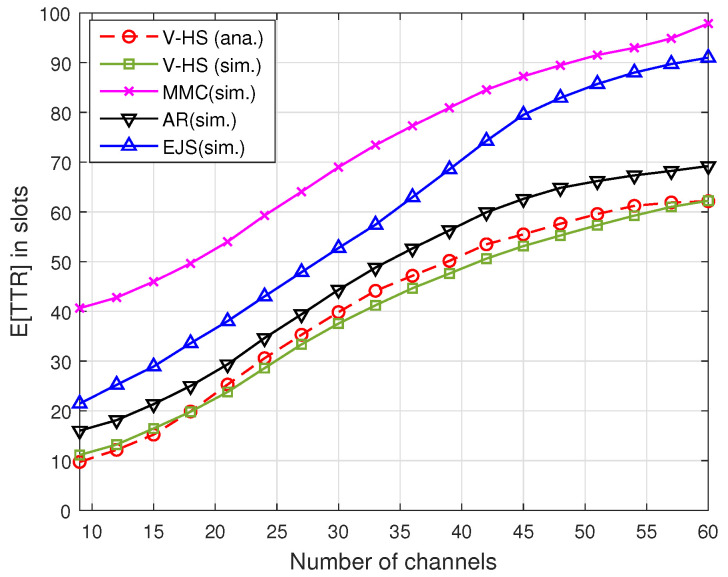
*E*[TTR] in symmetric model.

**Figure 14:**
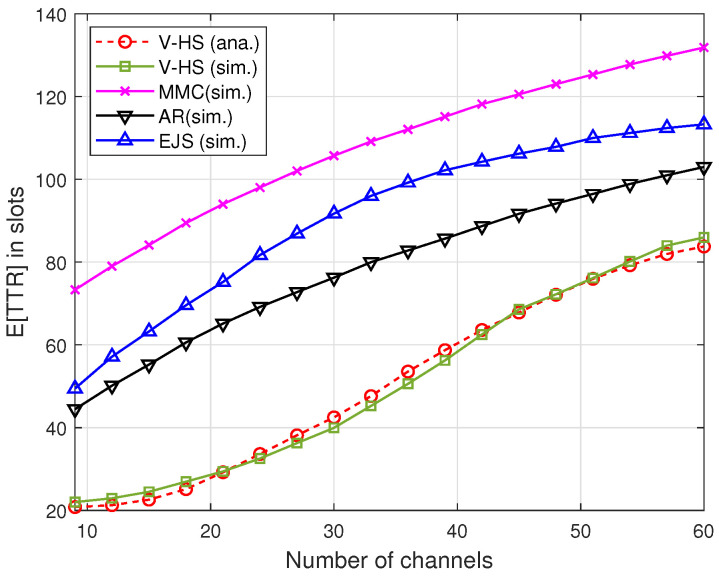
*E*[TTR] in asymmetric model.

Figure 13 and Figure 14 illustrate the TTR of the present scheme without MAC, where V-HS is compared with MMC, EJS, and AR for symmetric and asymmetric models, respectively, as a function of the number of channels. As shown in Figure 14, *G* was considered to be 60%, that is, the users have 60% of channels common between them. These results indicate that V-HS achieves reduced TTR compared to the other schemes for both symmetric and asymmetric models. MMC chooses a random prime number for the rendezvous operation, and in our simulation users select the prime repeatedly until rendezvous is achieved. In the case of JS and AR, a user stays for a long time in the stay period, which is *P* in every 3P, which increases the expected TTR, whereas V-HS takes advantage of odd and even slot structure, thus reducing the overall TTR as proved in our theoretical analysis. The simulation results also justify the theoretical *E*[TTR]. It can be observed in both figures that *E*[TTR] increases with the increase in the number of channels because the users have to hop on more channels.

**Figure 15:**
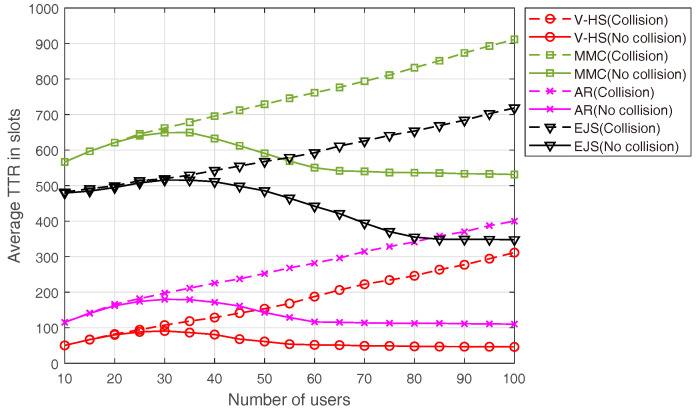
Average TTR in asymmetric mode.

Figure 15 depicts a comparison of the performance with the number of users in the asymmetric model. We have omitted the symmetric model here because its results are obvious from the results of the asymmetric model. In this case, all the schemes demonstrate a stable behavior with the increasing number of users when there is no collision. MMC, EJS, and AR are outperformed by V-HS because of the better hopping sequence, as analyzed earlier. However, with collision, the average TTR continues to grow linearly with the number of users, which specifies a major drawback of the hopping sequences in a real environment. Increasing the number of users means more users will hop on the same channel; however, under no collision, the trend is different than the time of collision, as the users will find each other on the same channel, but there is no probe request/response collision.

**Figure 16:**
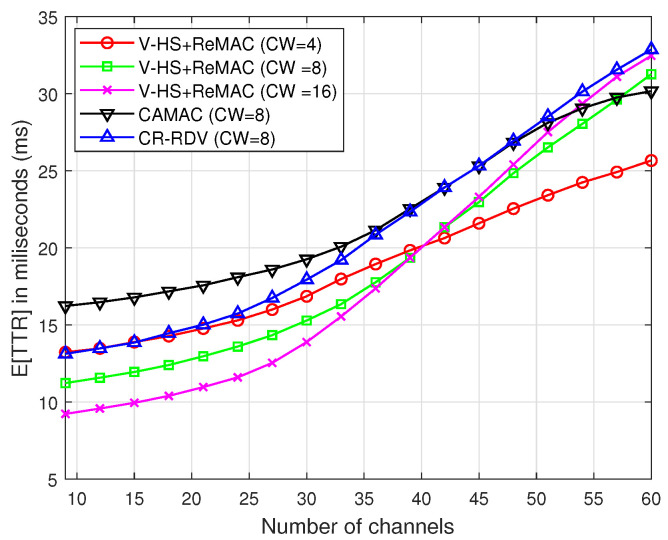
*E*[TTR] of ReMAC in the symmetric model(without multiple rendezvous opportunities).

**Figure 17:**
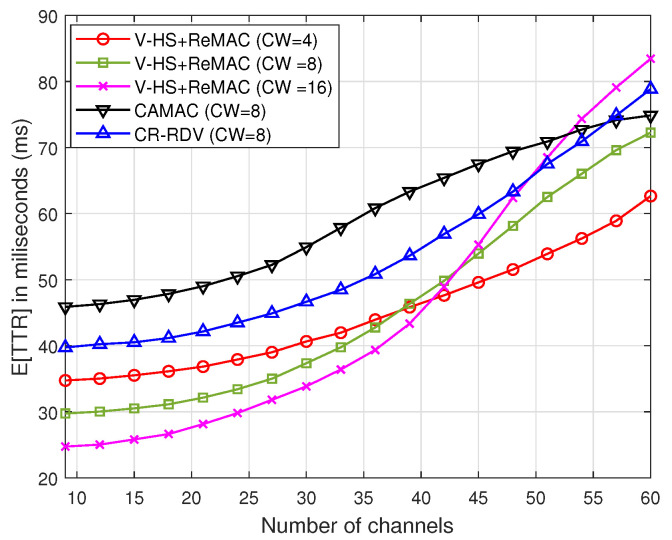
*E*[TTR] of ReMAC in the asymmetric model(without multiple rendezvous opportunities).

Next, the proposed ReMAC is integrated with the V-HS for both the symmetric and asymmetric models. To separate the effect of multiple rendezvous opportunities from ReMAC, we assume no retransmission in each slot; that is, if a collision occurs on the probe request or response, the user will hop to the next channel without retransmitting. Figure 16 and Figure 17 demonstrate a significant performance improvement by the integration of ReMAC and V-HS. The proposed ReMAC enhances a lower TTR compared to CAMAC and CR-RDV. CAMAC requires time synchronization before the rendezvous process, which leads to a higher TTR. In the case of CR-RDV, there is no process to reduce the overhead of RTS/RTS and CTS/CTS collisions. Therefore, some slots are wasted although the users are on the same channel. In ReMAC, users attempt to send at least one probe request before the next hop. The increase in the number of channels causes the SUs to be more distributed among channels. In both symmetric and asymmetric models, the TTR of CW=16 is better when the channel number is low. With a higher CW, the probability of collision is reduced. However, when the channel number increases, the TTR naturally increases, as shown in Figure 13 and Figure 14. In ReMAC, more channels have a better performance with CW=4. Both these figures verify that MAC has a strong impact on the TTR performance.

**Figure 18:**
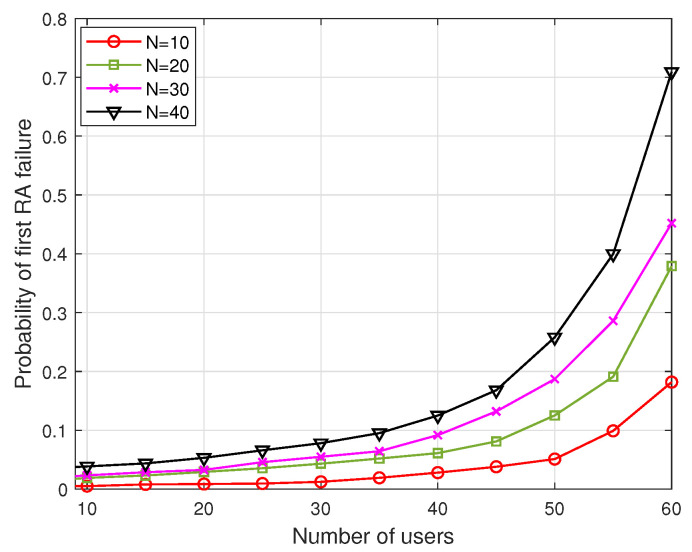
Probability of failure in first RA when CW=8.

**Figure 19:**
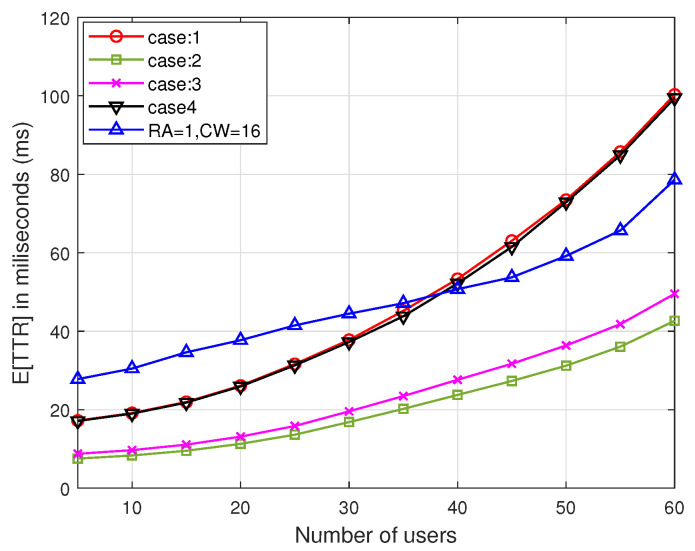
*E*[TTR] of multi-user ReMAC integrated with V-HS for various CW parameters.

Figure 18 depicts the probability of rendezvous failure at the first RA. With the increase in the number of users or with a decrease in the number of channels, failures occurrence increases because of the collisions among the probe messages. This result provides the advantage of exploiting multiple rendezvous opportunities in a slot. Figure 19 shows the obtained TTR of ReMAC for various combinations of $CW_{min}$, $CW_{max}$, and $RA_{max}$, which are listed in Table 4, where the number of channels is fixed to 20. Here, ReMAC adopts multiple rendezvous opportunities in a slot and an adaptive window size. Another simple case with RA=1 and CW=16 (here, CW is always fixed) is also compared to understand the effects of RA and CW. It can be observed that the TTR for cases 2 and 3 is considerably better than that of the other cases. For cases 1 and 4, the performance does not degrade significantly when the number of users is small. When the number of users increases, especially exceeding 25, the TTR for cases 1 and 4 increases and it is even worse than that of the simple case. This is because, for case 1, most users set a small initial CW, thereby always ending up with a collision. Although case 4 has a higher $CW_{max}$, it has only two RA opportunities during a slot. For these reasons, cases 2 and 3 outperform the other cases. From the results, ReMAC has been verified to work well with the features of adaptive collision resolution and multiple rendezvous opportunities, as well as the integration with V-HS.

**Figure 20:**
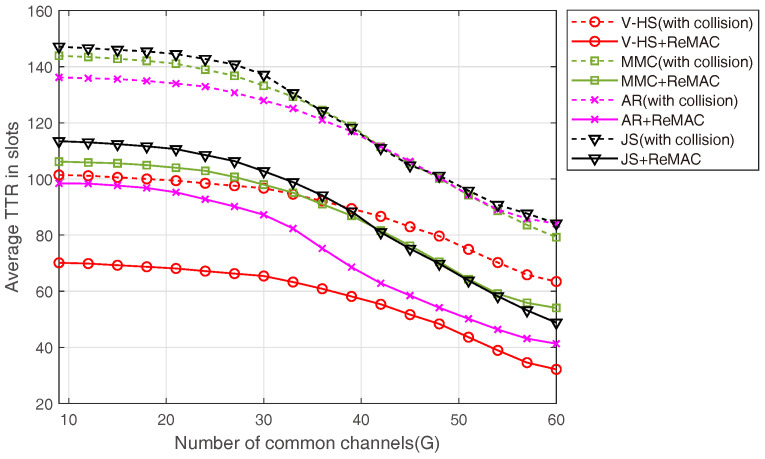
ReMAC with different number of common channels.

**Figure 21:**
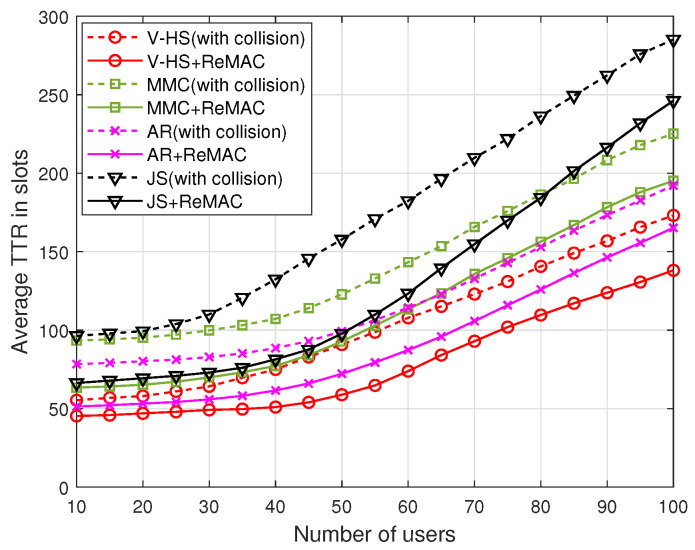
ReMAC with different number of users.

One of the major advantages of ReMAC is its interoperability. ReMAC works with any channel-hopping algorithm. Figure 20 presents the performance of ReMAC with MMC, EJS, AR, and the proposed V-HS. To signify the performance of ReMAC, we also present the TTR performance of all these CH sequences considering collision. With the increasing number of *G*, all the schemes achieve rendezvous at a lower TTR; however, this can be significantly reduced by ReMAC. In ReMAC, whenever a collision occurs, the user assumes that some users are trying to communicate on the channel. Therefore, the user modifies its backoff time and attempts to rendezvous again on the current channel. The simulation results presented in Figure 21 depict the average TTR for different CH schemes with respect to the increasing number of users. The average TTR increases with the increase in the number of users because it causes more collisions during rendezvous attempts. As explained earlier, ReMAC attempts to rendezvous on the channel based on $RA_{max}$.

## 7. Conclusions

In this paper, we propose a practical solution for the rendezvous problem. Our goal is directed to a twofold achievement: first, a fast hopping sequence, namely V-HS and second, ReMAC to avoid collision during the rendezvous process. In V-HS, during the even time slots, SUs hop on different channels, whereas during the odd time slots, SUs stay on the same channel for 2P. For the symmetric model where SUs see the same channels, V-HS guarantees rendezvous within 2P, which is less than that of the existing schemes, and for the asymmetric model, within 4P(P−G+1), which is not guaranteed in the existing schemes. In addition, based on probe messages, we propose a new rendezvous MAC protocol, ReMAC, that resolves the collision problems of the probe messages. In addition, the rendezvous opportunity increases owing to our design of multiple rendezvous attempts in a slot. The simulation results confirm that our proposed scheme enhances the TTR performance from the perspective of the channel-hopping sequence as well as the MAC protocol.

In the future, we plan to integrate the data exchange analysis for our proposed ReMAC. To the best of our knowledge, energy-efficient rendezvous is still an open area for research that we intend to explore. Finally, the free-for-all models allow malicious users to disrupt the rendezvous process. In this aspect, the current trends of rendezvous schemes need to be investigated further to make them applicable in real environments.

## Figures and Tables

**Table 1:** Arithmetic rendezvous algorithms.

Algorithm	Symmetric	Asymmetric	Collision
SSCH [18]	✓	✗	No
RingWalk [19]	✗	✓	No
AMRCC [20]	✓	✓	No
CRSEQ [21]	✓	✓	No
DRSEQ [22]	✓	✗	No
GOS [6]	✓	✗	No
MC [3]	✓	✗	No
MMC [3]	✗	✓	No
A-QCH [23]	✓	✗	No
JS [10]	✓	✓	No
EJS [24]	✗	✓	No
E-AHW [25]	✓	✓	No
T-CH/D-CH [26]	✓	✗	No
RCCH [27]	✓	✓	No
DSCR [28]	✓	✓	No
AR [9]	✓	✓	No
SAsync/AAsync [29]	✓	✓	No
OOC-CH [30]	✗	✓	No
CM2P-CH & CM4P-CH [31]	✓	✓	No
K-RCH [32]	✓	✗	No
V-CH (Proposed)	✓	✓	Yes

**Table 2:** Rendezvous MAC for wireless environment.

Protocol Name	Synchronization	Mechanism	Collision
COMAC [44]	✓	RTS/CTS	No
AR-CSMA/CA [45]	✗	RTS/CTS	No
CA-MAC [46]	✓	-	No
PSA [47]	✗	RTS/CTS	Yes
Slot Asyn. MAC [13]	✗	RTS/CTS	Yes
CoCH CSMA/CA [48]	✗	RTS/CTS	Yes
CR-RDV [49]	✗	RTS/CTS	Yes
ReMAC (Proposed)	✗	Probe request/response	Yes

**Table 3:** Protocol parameters of MAC and PHY layer.

Number of PUs	40
Number of SUs	60
Simulation area	100 m × 100 m
Data rate	1 Mbps
DIFS	50 μs
SIFS	10 μs
Probe request	40 bytes
Probe response	63 bytes
ACK	14 bytes
mini-slottime	20 μs

**Table 4:** Cases for simulation in Figure 19.

	${\mathrm{CW}}_{\min}$	${\mathrm{CW}}_{\max}$	${\mathrm{RA}}_{\max}$
case 1	2	16	4
case 2	4	32	4
case 3	8	32	3
case 4	10	20	2

## Data Availability

Not applicable.
